# High-Velocity Penetrating Pelvic Injury With Iron Rod: A Case Report and Literature Review

**DOI:** 10.7759/cureus.107886

**Published:** 2026-04-28

**Authors:** Vijendra D Chauhan, Faiz Akbar Siddiqui

**Affiliations:** 1 Department of Orthopaedics, Himalayan Institute of Medical Sciences, Dehradun, IND

**Keywords:** foreign body extraction, impalement trauma, multidisciplinary management, penetrating pelvic injury, road traffic accident

## Abstract

Penetrating pelvic impalement injuries caused by foreign objects are uncommon and potentially life-threatening due to the risk of severe hemorrhage and multi-organ involvement. We report the case of a 16-year-old female who sustained a penetrating injury when an iron rod/metal rod (handle-like structure) pierced her right gluteal region following a high-velocity road traffic collision. Initial assessment revealed a protruding metal object without active bleeding, and the patient was hemodynamically stable. Preoperative imaging demonstrated the trajectory of the foreign body through the gluteal musculature, penetrating the right iliac blade without involvement of the sacroiliac joint or acetabulum. A multidisciplinary surgical team performed a controlled exploratory laparotomy and removal of the object through a modified iliofemoral approach, with careful debridement and copious irrigation. No major neurovascular or visceral injuries were identified. The postoperative course was uneventful, and the patient was discharged on day 21 with no functional deficits.

This case highlights the importance of rapid assessment, meticulous pre-hospital handling, and avoidance of premature removal of impaled objects, which may maintain tamponade and prevent catastrophic bleeding. Definitive extraction should always occur under direct vision in a controlled operative environment. The successful outcome in this case is attributable to early resuscitation, detailed radiological evaluation, strategic surgical planning, and coordinated multidisciplinary involvement. Reporting such rare injuries contributes to improved awareness, clinical decision-making, and evolving management strategies. Early recognition and timely, organized intervention remain crucial to reducing morbidity and mortality in penetrating pelvic trauma.

## Introduction

Foreign bodies that strike the body with long, rigid edges can produce serious trauma, making management from initial hospitalization through surgical removal and follow-up care highly demanding [1]. Impalement injuries involve penetration by objects such as rods or poles and may completely perforate major anatomical structures. These injuries carry high morbidity and mortality because of the risk of complex injury patterns and massive pelvic bleeding [2].

Penetrating trauma to the pelvis is uncommon but particularly dangerous because of the close proximity of major vascular, urogenital, and gastrointestinal structures. Within this confined anatomical space, multiple vital organs are vulnerable to damage. The degree of organ involvement, the amount of blood loss, and the effectiveness of resuscitation become crucial determinants of overall outcome. These injuries frequently present with hemodynamic instability and demand rapid assessment and urgent surgical intervention. Laparotomy remains central to management, enabling early identification and treatment of associated injuries, and its success depends on planning the operative approach according to the trajectory of the impaling object [3,4].

Major pelvic injuries are usually associated with high-energy mechanisms such as road traffic collisions, pedestrian accidents, falls from height, or crush trauma [5]. Pelvic fractures constitute 3%-6% of all fractures in adults and appear in up to 20% of polytrauma cases. The incidence of pelvic fracture following blunt trauma ranges from 5% to 11.9%, with obese patients more likely to sustain such injuries than non-obese individuals [6].

Penetrating pelvic fractures, however, are far less common. Open pelvic fractures represent only 2.7%-4% of all pelvic fractures, and impalement injuries from steel bars are particularly rare compared with penetrating injuries involving the extremities. When visceral organs are affected, morbidity increases significantly, and mortality risk is high [1,2,7-9].

Early and effective resuscitation, precise assessment of associated injuries, and a coordinated multidisciplinary approach from pre-hospital care through definitive treatment are essential for improving survival and functional outcomes. The aim of the present case report is to describe a rare penetrating pelvic injury caused by an iron rod, highlight the multidisciplinary management that led to a successful outcome, and review the relevant literature to emphasize key considerations for timely diagnosis and surgical intervention.

## Case presentation

A 16-year-old female was traveling toward Rishikesh in a three-wheeler auto rickshaw when it collided head-on with a speeding car. During the impact, an iron rod/metal rod (handle-like structure) penetrated her right gluteal region, became lodged, and rendered her unable to move. Local bystanders cut the exposed portion of the object with a tool to extricate her from the vehicle, after which she was transported to the emergency department of the Himalayan Institute of Medical Sciences. On arrival, she was conscious, oriented, and hemodynamically stable with a blood pressure of 110/70 mmHg, pulse rate of 130/min, and oxygen saturation of 99%. Examination revealed a 2.5 cm-diameter metallic foreign body protruding from the right gluteal area with an 8 × 2.5 cm laceration and no active bleeding. Multiple abrasions were present over the right iliac fossa, and the proximal end of the object was palpable. Initial wound care included stabilization and sterile dressings over both the posterior entry site and the area of tenting near the right anterior superior iliac spine.

**Figure 1:**
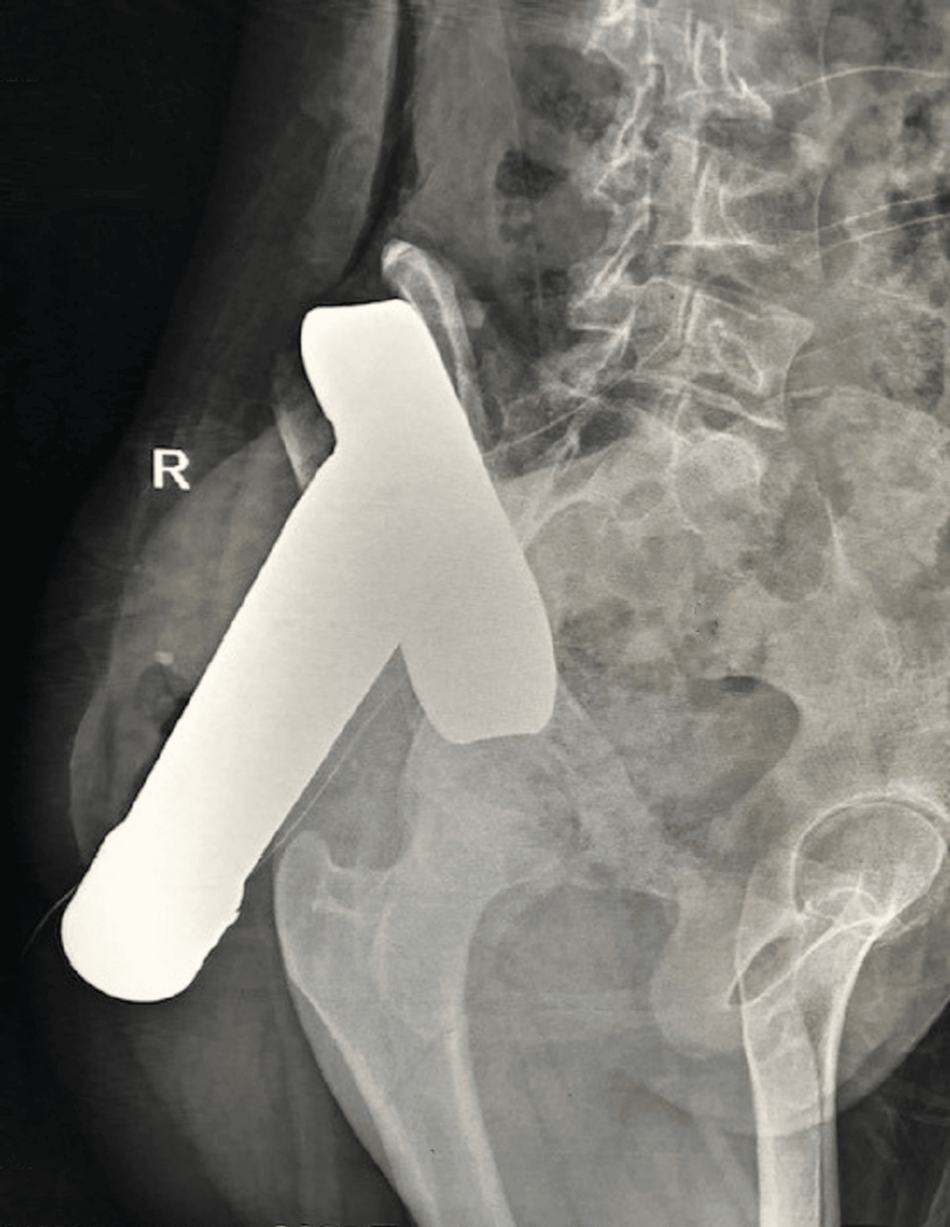
X-ray scan of the abdomen and pelvis demonstrating the trajectory of the metallic foreign body. The rod is seen passing through the right gluteal region and penetrating the right iliac blade without breaching the sacroiliac joint or acetabulum.

**Figure 2:**
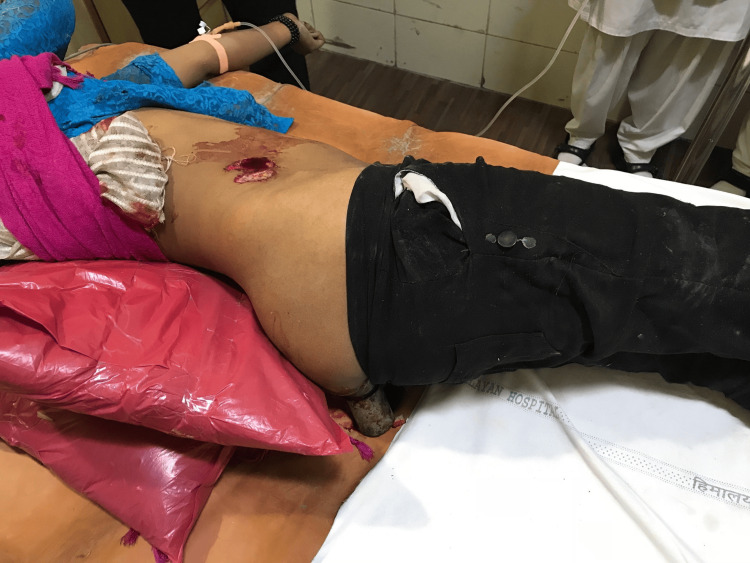
Clinical photograph on arrival showing the visible cut end of the metallic foreign body protruding through the right posterior gluteal region, with a contaminated soft-tissue wound and surrounding abrasions. The object is immobilized, and temporary sterile dressings have been applied before operative planning.

On presentation, the trauma team, including orthopedic and general surgeons, performed a rapid assessment. Radiographic evaluation with an X-ray of the abdomen and pelvis demonstrated the path of the metal rod passing through the gluteal musculature and penetrating the right iliac blade, without involvement of the sacroiliac joint or acetabulum (Figure 1). Clinically, only the cut end of the object was visible posteriorly, with a firm structure palpable in the right iliac fossa (Figure 2).

**Figure 3:**
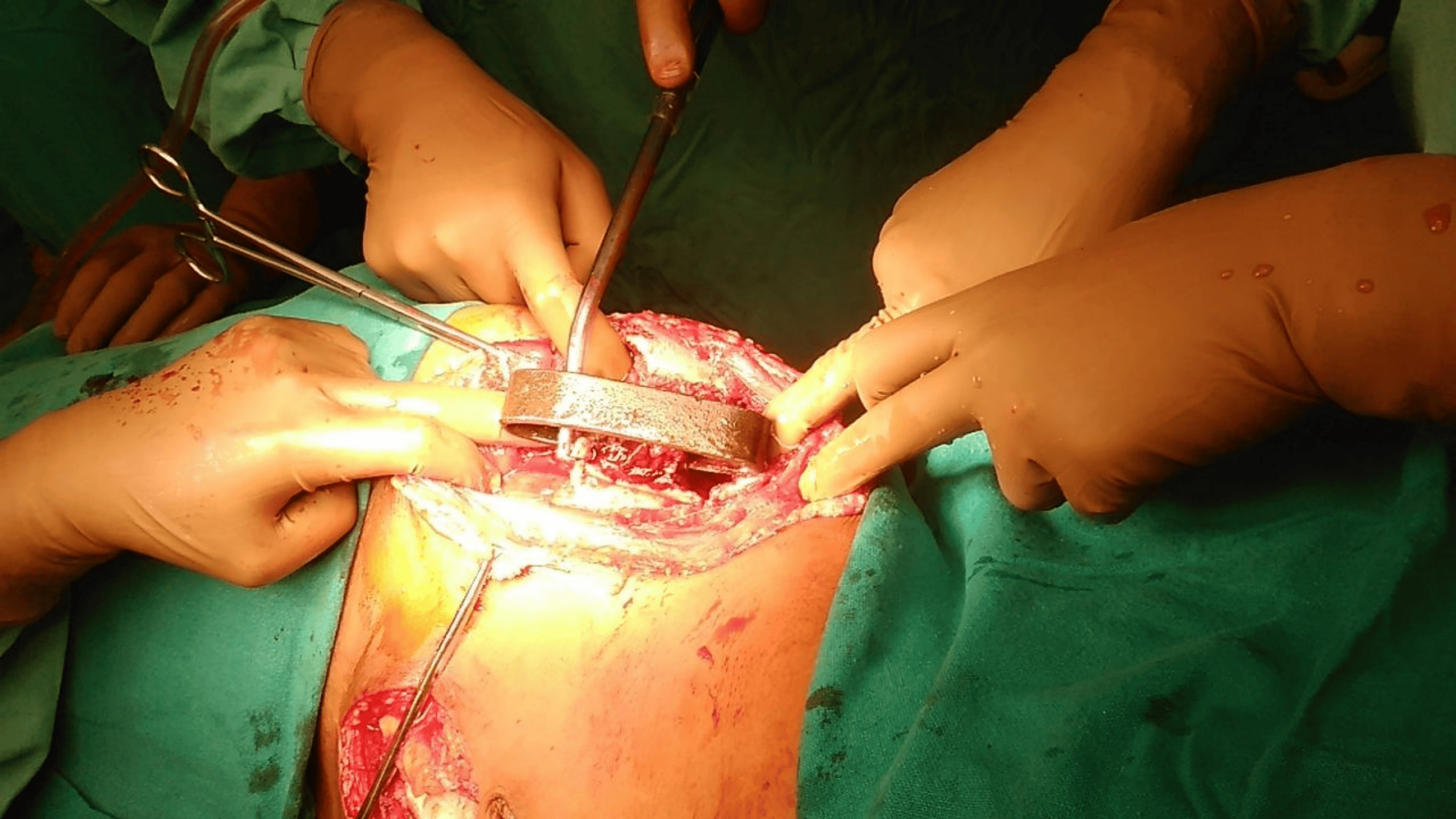
Intraoperative photograph showing exposure of the penetrating metallic foreign body through a modified iliofemoral approach. Multiple surgical retractors are used to visualize the track of the object along the iliac blade, allowing controlled mobilization and planning of safe extraction.

An indwelling urinary catheter was inserted, tetanus prophylaxis was administered, and a single intravenous dose of 1.5 g cefuroxime was given before transferring the patient to the operating room. Surgery was undertaken by a team consisting of orthopedic and general surgeons, with vascular and urology specialists on standby for any unexpected intraoperative findings. After a thorough assessment of the lower abdomen, an exploratory laparotomy was carried out to rule out visceral injury. Subsequently, a skin incision resembling a modified iliofemoral approach was made to access the trajectory of the foreign body (Figure 3).

**Figure 4:**
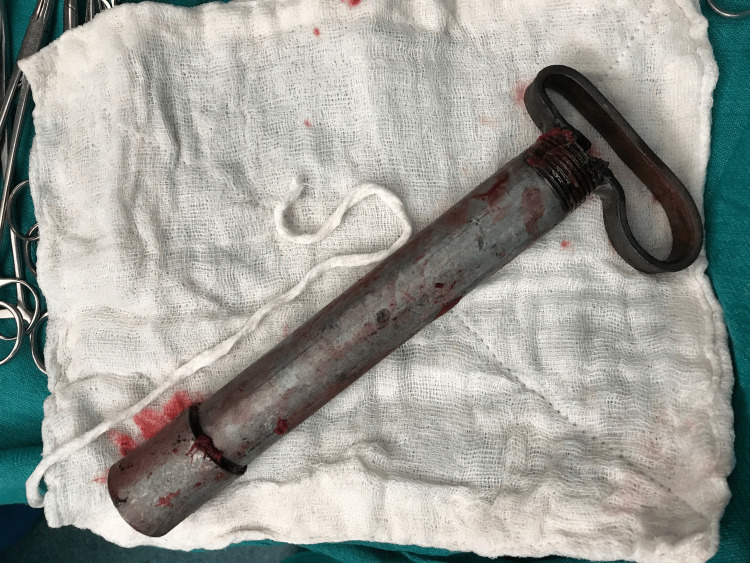
Extracted metallic foreign body after controlled surgical removal. The iron rod/metal rod (handle-like structure), previously impaled through the right gluteal region and iliac blade, is shown intact following debridement and removal under direct visualization.

With the patient positioned in an unconventional left lateral posture, the incision extended laterally from the anterior superior iliac spine and continued approximately 15 cm distally along the medial border of the iliac blade. Deeper exposure required a sharp detachment of the lateral abdominal wall muscles from the fractured edge of the iliac crest. The foreign body was visualized within the iliacus muscle, without proximity to the femoral neurovascular structures. Care was taken to preserve the lateral femoral cutaneous nerve, and the incision was lengthened distally along the iliac blade to connect the entry and exit wounds, allowing full visualization of the tract. After circumferential dissection and thorough debridement of both wounds, the metal rod was gently mobilized and extracted under direct vision (Figure 4).

**Figure 5:**
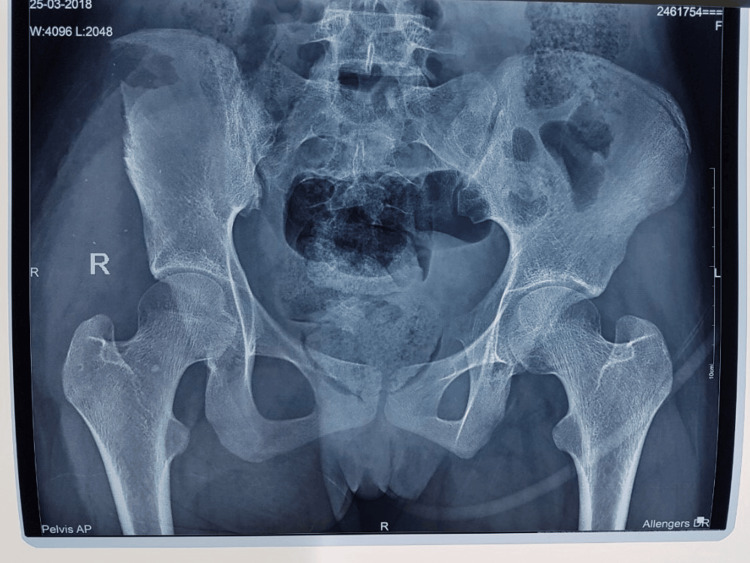
Postoperative anteroposterior pelvic radiograph showing intact bony alignment following removal of the penetrating metallic foreign body. No disruption of the sacroiliac joint or acetabular surfaces is evident, and no residual foreign material is visible.

After thorough debridement, the iliac blade was irrigated extensively with normal saline, and several small bone fragments were removed. Posterior wound inspection suggested that the rod had likely entered at this site and then tracked beneath the skin along the lateral border of the anterior superior iliac spine. Further debridement and generous lavage were completed without significant hemorrhage. A vacuum drain was placed prior to layered muscle closure, followed by definitive skin closure using dynamic tension techniques. Postoperative pelvic radiography demonstrated intact bony structures, with the sacroiliac joint and acetabulum unaffected and no retained foreign material (Figure 5). The patient’s recovery was uneventful, and she was discharged on postoperative day 21.

## Discussion

Perineal impalement injuries are severe events with substantial risk. The present case involves a high-velocity penetrating trauma to the pelvis, representing one of the most demanding situations encountered in trauma care. Such injuries commonly affect multiple structures, including soft tissues, bony pelvis, genitourinary organs, major vessels, and intra-abdominal viscera. Neurovascular and visceral damage often occur together in pelvic or abdominal impalement scenarios. Shrestha et al. previously described a similar case involving perineal penetration by an iron rod, although in their report the injury resulted from a fall onto the projecting object rather than a high-velocity impact [3]. Other etiologies include physical assaults and sexual abuse [10,11].

Up to one-quarter of affected individuals may succumb either en route to medical care or shortly after the injury because of uncontrollable bleeding [12]. Even in patients who respond to aggressive resuscitation and achieve hemostasis, septic complications remain a major concern, occurring in as many as 80% of cases involving perineal injuries [13]. Therefore, patient outcomes largely depend on the extent of organ damage, the volume of hemorrhage, and the effectiveness of resuscitation, together with timely control of infectious complications [10]. Moreover, available reports suggest that children tend to have better outcomes than adults when sustaining these types of injuries [14]. In this case, the patient made an uncomplicated recovery, likely because the foreign body followed a relatively straightforward path and did not damage any major intrapelvic structures. Injuries of this nature can potentially involve the iliac vessels, sacral plexus, sciatic nerve, female reproductive organs, and the femoral or popliteal neurovascular bundles either at the time of impact or during extraction of the object.

Pre-hospital management plays a crucial role in improving survival in such cases. Because the penetrating object can create a tamponade effect, limiting hemorrhage in the injured tissues, it should not be withdrawn until the patient is in a controlled setting. Removal must be performed under direct visualization in the operating room to reduce the risk of catastrophic bleeding [15]. If necessary, the object may be trimmed to allow safer transport, but it must be stabilized to prevent movement. Securing the impaling object helps avoid additional soft-tissue injury and minimizes the risk of further bleeding [11]. In our case, the iron rod/metal rod (handle-like structure) penetrated the gluteal region, became firmly lodged, and rendered the patient unable to move. Local residents cut the exposed portion of the iron rod/metal rod with a tool to free her from the three-wheeler. Shrestha et al. similarly reported cutting the projecting iron rod with minimal manipulation to allow transport, with no effort to remove the object until the patient reached the operating room [3].

Because of the complexity and rarity of these injuries, standardized treatment protocols are lacking. Careful evaluation and preoperative planning, including selection of the surgical approach and patient positioning on the operating table, are crucial when removing deeply embedded foreign objects. An inappropriate incision in anatomically intricate regions such as the pelvis or abdomen can limit visualization, making extraction difficult or even unachievable. In our case, an X-ray scan of the abdomen and pelvis helped delineate the path of the metal object, showing its course through the gluteal region and penetration of the right iliac blade, without involvement of the sacroiliac joint or acetabulum of the right hemipelvis.

The strength of this report lies in detailed clinical documentation, multidisciplinary management, and the successful outcome of a rare penetrating pelvic injury. As a single case, it is limited by a lack of generalizability and the absence of long-term functional follow-up. Larger series or pooled analyses are needed to better define standardized protocols. We recommend prompt imaging, controlled extraction under direct visualization, and coordination between trauma, orthopedic, vascular, and general surgery teams. The key take-home message is that timely, well-planned intervention can significantly reduce morbidity and prevent fatal complications in penetrating pelvic impalement injuries.

## Conclusions

Penetrating pelvic injuries caused by impalement with foreign objects are rare but potentially life-threatening due to the high risk of vascular, neurological, and visceral involvement. Early recognition, meticulous pre-hospital care, and timely multidisciplinary management are critical to achieving favorable outcomes. Preoperative imaging, thoughtful surgical planning, and controlled removal of the object under direct visualization are essential steps in avoiding catastrophic complications. This case demonstrates that even severe penetrating pelvic trauma can result in full recovery when managed appropriately. Continued reporting of such cases will improve understanding, support clinical decision-making, and refine treatment strategies for similar injuries in the future.
